# Hydrogen sulfide signaling in plant adaptations to adverse conditions: molecular mechanisms

**DOI:** 10.1093/jxb/erab239

**Published:** 2021-06-02

**Authors:** Angeles Aroca, Jing Zhang, Yanjie Xie, Luis C Romero, Cecilia Gotor

**Affiliations:** 1 Instituto de Bioquímica Vegetal y Fotosíntesis, Consejo Superior de Investigaciones Científicas and Universidad de Sevilla, Avenida Américo Vespucio, 49, 41092 Seville, Spain; 2 Laboratory Center of Life Sciences, College of Life Sciences, Nanjing Agricultural University, Nanjing, PR China; 3 VIB-Ugent, Belgium

**Keywords:** Abscisic acid, persulfidation, proteomics, redox modifications, stomatal movement, sulfenylation

## Abstract

Hydrogen sulfide (H_2_S) is a signaling molecule that regulates critical processes and allows plants to adapt to adverse conditions. The molecular mechanism underlying H_2_S action relies on its chemical reactivity, and the most-well characterized mechanism is persulfidation, which involves the modification of protein thiol groups, resulting in the formation of persulfide groups. This modification causes a change of protein function, altering catalytic activity or intracellular location and inducing important physiological effects. H_2_S cannot react directly with thiols but instead can react with oxidized cysteine residues; therefore, H_2_O_2_ signaling through sulfenylation is required for persulfidation. A comparative study performed in this review reveals 82% identity between sulfenylome and persulfidome. With regard to abscisic acid (ABA) signaling, widespread evidence shows an interconnection between H_2_S and ABA in the plant response to environmental stress. Proteomic analyses have revealed persulfidation of several proteins involved in the ABA signaling network and have shown that persulfidation is triggered in response to ABA. In guard cells, a complex interaction of H_2_S and ABA signaling has also been described, and the persulfidation of specific signaling components seems to be the underlying mechanism.

## Teaser

This review focuses on recent advances relating to H_2_S signaling mechanisms, and highlights interconnections between H_2_S and H_2_O_2_ at the post-translational modification level and with ABA in stomatal movement.

## Introduction

Hydrogen sulfide (H_2_S) is a colorless gas with a characteristic unpleasant odor. In nature, H_2_S is present in volcanic gas, hot springs, rock salts, and natural gas, as well as in emissions produced as a result of industrial activity. In biological systems, H_2_S can be considered an ancient molecule since it originates from bacterial anaerobic metabolism. In the absence of oxygen, sulfur-reducing microorganisms use different forms of oxidized sulfur as electron acceptors during the degradation of simple organic matter, producing H_2_S and CO_2_ (Offre *et al.*, 2013). H_2_S is additionally used by sulfur-oxidizing bacteria as an electron donor in anoxygenic photosynthesis to produce oxidized sulfur compounds (Johnston *et al.*, 2009).

H_2_S has long been considered a toxic molecule dangerous to the environment and complex biological organisms. In mammals, the presence of sulfide in mitochondria causes the inhibition of cytochrome *c* oxidase of the respiratory chain, as does the presence of carbon monoxide (CO) and nitric oxide (NO) (Cooper and Brown, 2008). However, below a specific concentration threshold, CO, NO, and H_2_S affect various cellular events and are currently considered to be signaling molecules that function as physiological gasotransmitters (Wang, 2014). In plants, H_2_S is also recognized to have the same relevance as other signaling molecules, such as NO and hydrogen peroxide (H_2_O_2_) (Calderwood and Kopriva, 2014; Aroca *et al.*, 2018; Aroca *et al.*, 2020). All these molecules, including H_2_S, show toxicity/signaling duality, depending on the concentration threshold.

Although H_2_S is known to be present in mammalian tissues, its intracellular production and signaling function as a neuromodulator were first established in the late 20th century (Abe and Kimura, 1996). H_2_S is produced endogenously by cells through different enzymes involved in cysteine metabolism, in both mammals and plants. In plants, H_2_S is also produced in the photosynthetic sulfate assimilation pathway in the chloroplast (Gotor *et al.*, 2019). Intensive research on H_2_S has been carried out in recent years both in animals and in plants, and an impressive exponential increase in the number of original publications and reviews has occurred. Consequently, the number of biological functions in which sulfide is known to be involved has rapidly increased. In plants, H_2_S has been shown to be essential in regulating a wide range of vital processes. H_2_S improves the tolerance and protection of plants to numerous adverse environmental conditions, and, in this way, it allows plant adaptability and viability, and its beneficial effects play a role in important aspects of development (Zhang *et al.*, 2021b; Zhou *et al.*, 2021b). Sulfide also regulates critical processes, including autophagy and abscisic acid (ABA)-dependent stomatal movement (Gotor *et al.*, 2019; Laureano-Marín *et al*., 2020; Zhang *et al.*, 2020). In addition, the interplay of H_2_S with other signaling molecules and phytohormones in multiple physiological processes has been extensively described (Aroca *et al.*, 2020).

Despite the very large number of plant studies that are continuously being conducted, studies on the molecular mechanisms through which sulfide exerts its regulatory effects are still scarce. We believe that this aspect deserves specific attention, and this review highlights the progress obtained in understanding the mechanism of action of sulfide in plant systems. The most recent outcomes on the mechanism of the sulfide control of guard cell ABA signaling are also highlighted.

## Hydrogen sulfide action

The reaction mechanism in which H_2_S participates and exerts regulatory and signaling function is complex, and it is necessary to take into account the complex reactivity of this molecule. H_2_S encompasses neutral H_2_S and anionic forms (hydrosulfide, HS^–^, and sulfide, S^2–^) with p*K*_a1_ and p*K*_a2_ values of 6.9 and >12, respectively (Kabil and Banerjee, 2010). Therefore, in aqueous solution, H_2_S exists in equilibrium with its H^+^ and HS^–^ anionic forms; these latter are unable to cross organelle membranes. Under physiological pH conditions, two-thirds of H_2_S exists in the form of HS^–^. However, the lipid solubility of H_2_S and its membrane permeability promote the biological distribution of sulfide species within cells (Cuevasanta *et al.*, 2012).

The mechanism of action of H_2_S is related to the characteristics of acid–base behavior and chemical reactivity with other biochemical molecules, such as low-molecular weight (LMW) thiols, protein thiols, protein metal centers, and biological oxidants. Among these oxidants, the hydroxyl radical (OH·), nitrogen dioxide (NO_2_·), superoxide radical (O_2_· ^–^), H_2_O_2_, peroxynitrite (ONOOH), and hypochlorite (HOCl) can support H_2_S oxidation (Li and Lancaster, 2013; Zaffagnini *et al.*, 2019).

Metalloproteins are well-established biochemical targets of H_2_S that covalently attach to heme porphyrins. Thus, H_2_S acts as a potent inhibitor of mitochondrial cytochrome *c* oxidase, inhibiting mitochondrial respiration, releasing cytochrome *c* during apoptosis, and stimulating procaspase 9 persulfidation (Vitvitsky *et al.*, 2018). H_2_S can also react quickly and reversibly with other ferric heme proteins such as methemoglobin and leghemoglobin to reduce their iron center and form a complex (Jensen and Fago, 2018; Boubeta *et al.*, 2020). In addition to modifying heme proteins, H_2_S can also modify Zn-finger proteins, but this leads to persulfidation and rapid thiol oxidation (Lange *et al.*, 2019).

A second mechanism of action of H_2_S that is well established in mammalian and plant systems is the modification of proteins by the oxidation of cysteine residues to form corresponding persulfides (Filipovic, 2015; Gotor *et al.*, 2019). Protein thiol persulfidation has been widely described for numerous proteins, and it was initially described as *S*-sulfhydration in mouse liver. Susceptibility of several proteins to modification by sulfide has been determined (Mustafa *et al.*, 2009; Filipovic *et al.*, 2018). In plants, three high-throughput proteomic analyses also revealed the presence of persulfidation in the Arabidopsis proteome, showing >3400 and 5214 proteins susceptible to persulfidation in leaf and root tissue, respectively (Aroca *et al.*, 2015, 2017*a*; Jurado-Flores *et al.*, 2021). Different studies on this post-translational modification of specific proteins have shown that it results in changes to the function of the proteins, altering their catalytic activity or intracellular location and inducing important physiological effects, ranging from regulation of autophagy, ABA-dependent stomatal closure, ethylene biosynthesis, and root hair growth, to resistance to oxidative stress (Table 1). Persulfides on specific cysteine residues have been described in different Arabidopsis proteins, including abscisic acid-insensitive 4 (ABI4) (Zhou *et al.*, 2021a), cytosolic ascorbate peroxidase 1 (APX1) (Aroca *et al.*, 2015), cytosolic glyceraldehyde-3-phosphate dehydrogenase (GapC1) (Aroca *et al.*, 2017*b*), actin 2 (ACT2) (Li *et al.*, 2018), l-cysteine desulfhydrase (DES1), respiratory burst oxidase homolog protein D (RBOHD) (Shen *et al.*, 2020), SNF1-related protein kinase SnRK2.6 (Chen *et al.*, 2020), and the autophagic proteins ATG4a and ATG18a (Laureano-Marín *et al*., 2020; Aroca *et al.*, 2021*a*). In addition, tomato 1-aminocyclopropane-1-carboxylic acid oxidases 1 and 2 (LeACO1 and LeACO2, respectively) (Jia *et al.*, 2018) and tomato antioxidant enzymes (Li *et al.*, 2020) have also been demonstrated to be persulfidated on specific cysteine residues (Table 1).

**Table 1.** Plant proteins persulfidated at specific cysteine residues

Protein	Accession number	No. of Cys residues	Persulfidated Cys	Effect of the modification	Reference
Arabidopsis abscisic acid insensitive 4 (ABI4)	At2g40220	3	Cys250	MAPKKK18 transactivation/increase the MAPK cascade signal in response to ABA	Zhou *et al.* (2021a)
Arabidopsis actin2 (ACT2)	At3g18780	4	Cys287	Inhibition of actin polymerization/depolymerization of F-actin bundles/inhibition of root hair growth	Li *et al.* (2018)
Arabidopsis cytosolic ascorbate peroxidase1 (APX1)	At1g07890	5	Cys32	Increase of enzyme activity	Aroca *et al.* (2015)
Arabidopsis autophagy-related protein cysteine protease 4a (ATG4a)	At2g44140	12	Cys170	Inhibition of proteolytic activity/repression of autophagy	Laureano-Marín *et al*., (2020)
Arabidopsis autophagy-related protein ATG18a	At3g62770	8	Cys103	Activation of ATG18a binding capacity to specific phospholipids/repression of auotphagy	Aroca *et al.* (2021a)
Arabidopsis l-cysteine desulfhydrase1 (DES1)	At5g28030	3	Cys44 and Cys205	Increase of enzyme activity/induction of H_2_S production/ABA-dependent stomatal closure	Shen *et al.* (2020)
Arabidopsis cytosolic glyceraldehyde 3-phosphate dehydrogenase C1 (GapC1)	At3g04120	2	Cys160	Enhanced nuclear localization	Aroca *et al.* (2017b)
Arabidopsis open stomata1/SNF1-related protein kinase2.6 (OST1/SnRK2.6)	At4g33950	6	Cys131 and Cys137	Increase of enzyme activity/enhanced interaction with ABA response factor ABF2/ABA-dependent stomatal closure	Chen *et al.* (2020)
Arabidopsis NADPH oxidase respiratory burst oxidase homolog protein D (RBOHD)	At5g47910	10	Cys825 and Cys890	Increase of enzyme activity/induction of H_2_O_2_ production/ABA-dependent stomatal closure	Shen *et al.* (2020)
Tomato 1-aminocyclopropane-1-carboxylic acid oxidases 1 and 2 (ACO1/2)	NP_001234024/NP_001316842	4	Cys60	Inhibition of enzyme activity/repression of ethylene biosynthesis	Jia *et al.* (2018)
Tomato cytosolic ascorbate peroxidase1 (cAPX1)	NP_001234782.1	6	Cys168	Increase of enzyme activity/enhanced resistance to oxidative stress	Li *et al.* (2020)
Tomato catalase1 (CAT1)	NP_001234827.1	10	Cys234	Inhibition of enzyme activity/enhanced resistance to oxidative stress	Li *et al.* (2020)
Tomato peroxidase 5 (POD5)	XP_004235031.1	10	Cys46 and Cys61	Increase of enzyme activity/enhanced resistance to oxidative stress	Li *et al.* (2020)

Shown as the accession number, the number of cysteine residues in the amino acid sequence, the specific persulfidated cysteines, and the effects and biological consequences of the post-translational modification of the proteins.

**Fig. 1.**
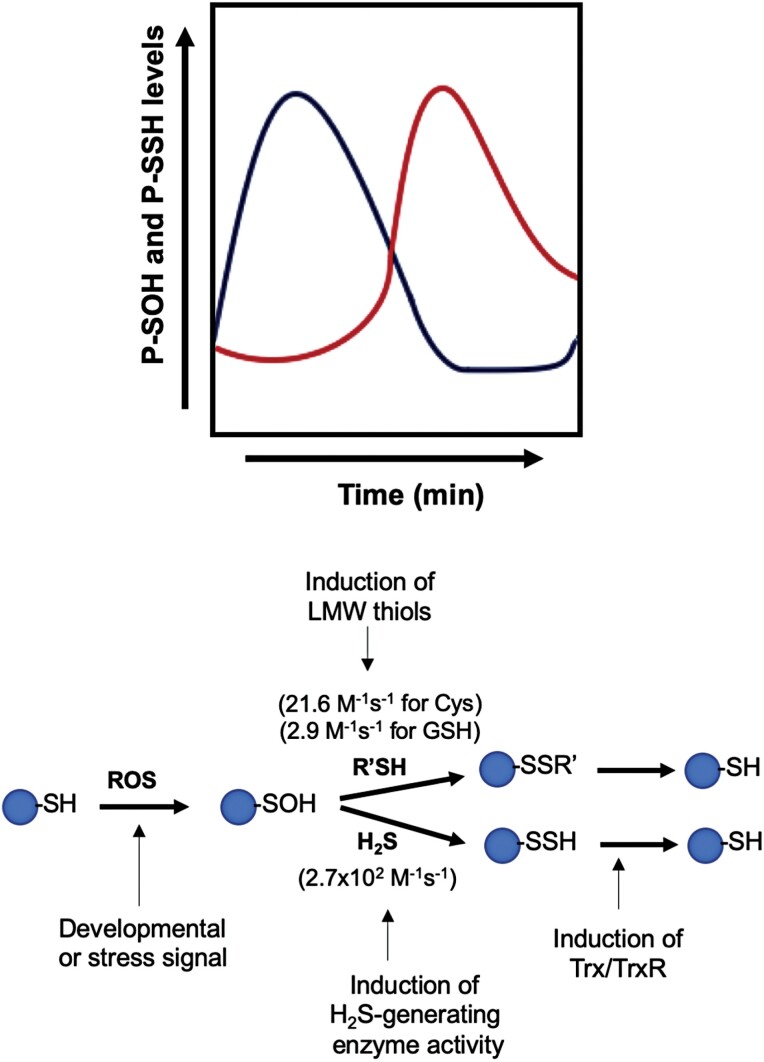
Schematic representation of the temporal dynamic of protein sulfenylation (P-SOH) and persulfidation (P-SSH) in different cell types (after Zivanovic *et al.*, 2019). After a transient ROS production induced by developmental or stress signals, the levels of sulfenylation in proteins are increased, accompanied by an increase in the activity of sulfide-generating enzymes and/or induction of low molecular weight (LMW) thiols, followed by a transient increase in protein persulfidation reversed by the action of reducing enzymes such as the thioredoxin system (Trx/TrxR). The rate constants for the reaction of R-SOH with LMW thiols and H_2_S at physiological pH 7.4 are shown (Cuevasanta *et al.*, 2015).

Numerous biochemical and genetic data have established beyond doubt the signaling effect of H_2_S in cells through persulfidation, with important consequences for numerous physiological and pathological processes in mammals and plants (Yuan *et al.*, 2017; Paul and Snyder, 2018; Aroca *et al.*, 2020). However, the precise mechanism that leads to the modification and the sulfur species that produces the protein persulfide formation is the subject of extensive debate and study. H_2_S, or its ionic forms, HS^–^ and S^2–^, cannot react directly with protein thiols and requires the presence of an oxidant. Thus, H_2_S can react with oxidized cysteine residues as sulfenic acid (R-SOH), but also with protein nitrosothiols (R-SNO) to give protein persulfides, but this latter process is thermodynamically unfavorable (Filipovic *et al.*, 2012). H_2_S can also chemically react with disulfides (R-S-S-R), but this seems unlikely to occur due to the low level of intracellular H_2_S and the slow reaction rate (Filipovic, 2015). Therefore, the reaction of H_2_S with protein sulfenic acid to form protein persulfide seems the most plausible explanation for H_2_S action. Cysteine residue oxidation represents a way for redox control of protein function and, therefore, H_2_O_2_ signaling takes place via the oxidation of cysteine to sulfenic acid, and the direct outcome on proteins is protein sulfenylation (Cuevasanta *et al.*, 2015; Zivanovic *et al.*, 2019; Willems *et al.*, 2020). Although sulfenylated residues (R-SOH) can be reversed to reduced thiol by the action of a diverse set of reducing enzymes, stress conditions can lead to the overoxidation of cysteine residues originating the sulfinic (-SO_2_H) or sulfonic (-SO_3_H) motifs that are irreversible (Zaffagnini *et al.*, 2019). However, in the catalytic cycle of peroxiredoxins, it was shown that the sulfinic cysteine can be reduced by sulfiredoxin in the presence of ATP via the formation of a phosphoryl intermediate (Sevilla *et al.*, 2015). Protein sulfenic acid residues can react either with LMW thiols or with H_2_S (HS^−^ ionic form), but the latter shows a significantly higher rate constant (Fig. 1). In fact, protein sulfenic residues react two orders of magnitude faster with H_2_S than with glutathione (Cuevasanta *et al.*, 2015). Since H_2_S reacts with sulfenylated residues to form persulfide, protein persulfidation may play a role in H_2_O_2_-based signal transduction by preventing the overoxidation of cysteine residues, resulting in the loss of protein function. Under persistent oxidation stress, persulfidated proteins can react with ROS to form perthiosulfenic acids (-SSOH) and, in the presence of excess oxidant, perthiosulfenic acid can be oxidized to perthiosulfinic (-SSO_2_H) and perthiosulfonic acid (-SSO_3_H) (Aroca *et al.*, 2018; Filipovic *et al.*, 2018). These oxidized perthiol residues can be reduced back to thiol by the action of glutathione and thioredoxin systems, as has been demonstrated in mouse liver (Zivanovic *et al.*, 2019; Dóka *et al*., 2020). The protective effect of persulfidation against overoxidation has also been shown in different cell types, where protein persulfidation increases following a phase-shifted curve after an increase in protein sulfenylation (Zivanovic *et al.*, 2019) (Fig. 1).

**Fig. 2.**
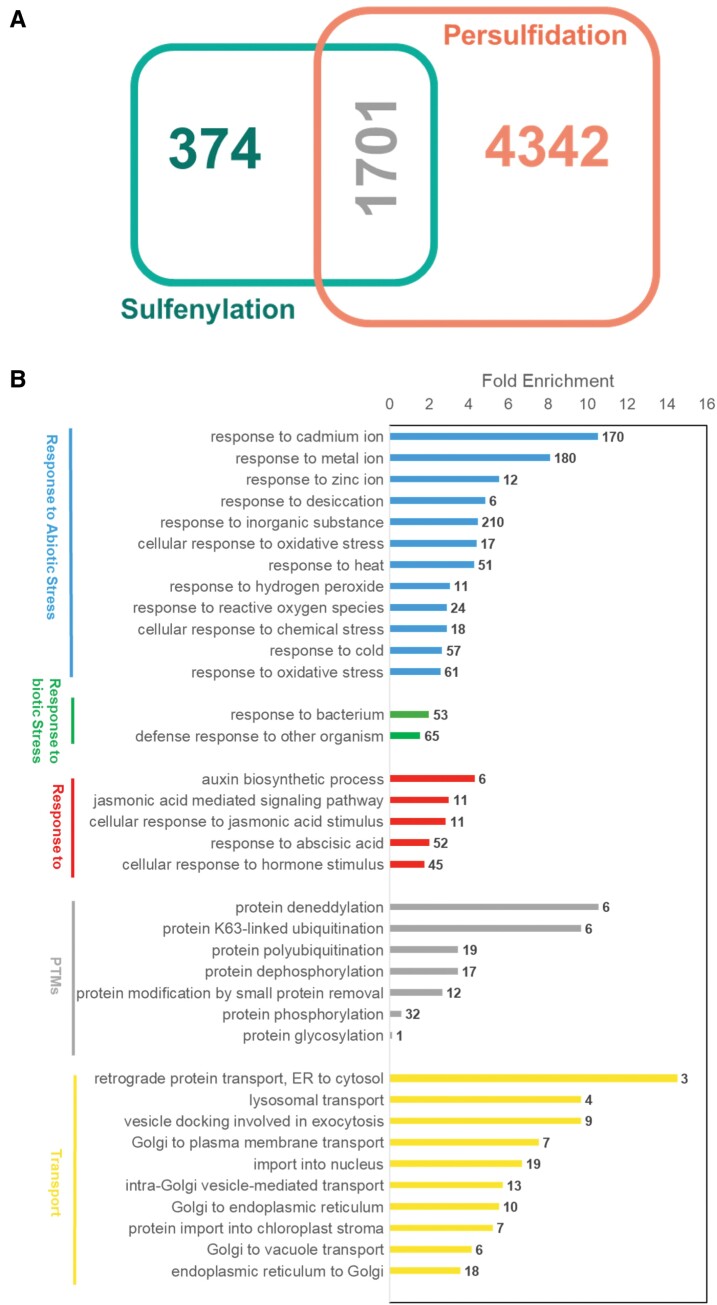
Comparison of persulfidated and sulfenylated proteins. (A) Venn diagram showing the number of proteins. (B) Fold change enrichment of GO terms of common proteins modified by sulfenylation and persulfidation. Analysis was performed with PANTHER software. The numbers beside the bars indicate the number of proteins associated with each GO term for the input set.

Redox regulation has been shown to be involved in many signaling processes that regulate environmental (biotic and abiotic) stress responses (Alvarez *et al.*, 2012; Hancock, 2019), development (Jia *et al.*, 2015; Deng *et al.*, 2020), or autophagy and cell death (Gotor *et al.*, 2013; Pérez-Pérez *et al*., 2014; Xie *et al.*, 2014), processes where H_2_S is also involved. There is a lot of evidence that there is an overlap between ROS and H_2_S; therefore, together, protein cysteine oxidation and persulfidation may represent a mechanism for the modulation of signaling processes induced by developmental or environmental stress events. In recent years, many proteins have been described as sulfenylation targets in Arabidopsis in several works, revealing >2000 targets for this modification (De Smet *et al.*, 2019; Huang *et al.*, 2019; Wei *et al.*, 2020). In a comparison performed between the sulfenylated and the previously identified persulfidated proteins, >6000 targets (Aroca *et al.*, 2015, 2017*a*; Laureano-Marín *et al*., 2020; Jurado-Flores *et al.*, 2021) revealed that 82% of the sulfenylated proteome described in Arabidopsis also undergo persulfidation (Fig. 2A). A total of 1701 proteins are targets for either sulfenylation or persulfidation, a number that must be underestimated taking into account that the Arabidopsis samples were very different in all these proteomic analyses. Despite the probable differences in their metabolism, the number of common proteins in both proteomes is considerably high, revealing the role of these modifications in the finely tuned balance between H_2_O_2_-based signal transduction and protection against overoxidation. Gene Ontology (GO) enrichment analysis of these proteins showed that several of these targets are associated with abiotic stress response-related GO terms, such as response to cadmium (170), metal ion (180), and zinc (12), response to oxidative stress (61), cellular response to oxidative stress (17), response to cold (57), response to heat (51), response to ROS (24), and response to H_2_O_2_ (11), among others (Fig. 2B). Included in these targets, three l-ascorbate peroxidases, four dehydroascorbate reductases, three glutaredoxins, 10 thioredoxins, two nitrate reductases, and numerous FAD/NAD(P)-oxidoreductases were found to be regulated by persulfidation and sulfenylation (see Table S1 at Zenodo https://zenodo.org/record/4727058), underlying the signaling role of these modifications in the activation of the antioxidant system against oxidative stress (Aroca *et al.*, 2021*b*). In addition, GO enrichment showed that among those proteins regulated by persulfidation and sulfenylation, targets involved in response to biotic stress, hormones, signaling, other post-translational modifications, and transport were found (Fig. 2B).

## Role of hydrogen sulfide in ABA signaling

In plants, precise mechanisms have been developed to perceive environmental stress. ABA, an important plant hormone, is involved in the regulation of growth and developmental processes, and defense against various environmental stresses. ABA is a central regulator that triggers complex signaling networks and is also involved in stomatal movement. Under certain conditions, ABA concentrations increase to activate these signaling pathways and, consequently, ABA binds to the ABA receptor protein family members Pyrabactin Resistance 1 (PYR1)/PYR1-Like (PYL)/Regulatory Component of ABA receptor (RCAR), and inhibits the activity of clade A protein phosphatases (PP2Cs) (Fujii *et al.*, 2009; Ma *et al.*, 2009; Park *et al.*, 2009). This process then results in the release of sucrose non-fermenting 1 (SNF1)-related protein kinase 2s (SnRK2s) from suppression by the PP2Cs, enabling the activation of SnRK2 protein kinases. These kinases subsequently phosphorylate and activate dozens of downstream targets (Hauser *et al.*, 2017).

Extensive and convincing evidence published in the last decade has shown a close inter-relationship between H_2_S and physiological processes regulated by the hormone ABA, suggesting that crosstalk occurs between both molecules in regulation and signaling in plants (Gotor *et al.*, 2019; Aroca *et al.*, 2020). It has been widely reported that H_2_S plays a role in stomatal closure, and the latest data are discussed in detail below. In addition to its role in plant growth and development, ABA plays a crucial role in plant responses to environmental stresses such as drought, salinity, osmotic stress, and heat stress, processes in which H_2_S has shown a protective effect, alleviating the oxidative stress associated with these adverse conditions (Gotor *et al.*, 2019). There is a large amount of additional evidence that interconnects H_2_S signaling with other plant processes regulated by ABA beyond mere antioxidant defenses. For example, it has been observed that the response to drought or heat mediated by ABA induces the accumulation of intracellular H_2_S, and exogenous H_2_S addition increases plant tolerance to these stresses (Jin *et al.*, 2011; Li and Jin, 2016). It has also been observed that ABA shows an opposite effect on the transcriptional regulation of the cytosolic l-cysteine desulfhydrase (DES1) that catalyzes the desulfuration of cysteine to generate H_2_S, depending on the tissue, inhibiting its transcription in mesophyll cells and increasing its transcription in guard cell-enriched tissues (Scuffi *et al.*, 2014). In general, sulfate availability affects the ABA content and germination response to ABA and salt stress, highlighting the importance of sulfur for stress tolerance (Cao *et al.*, 2014). From a molecular point of view, the most extensive proteomic analyses published to date on protein persulfidation has shown that several proteins involved in ABA signaling, such as PYR1 and PYL, SnRK2.2 protein kinase, and the protein phosphatase HAB2, are capable of being persulfidated (Aroca *et al.*, 2017*a*; Jurado-Flores *et al.*, 2021).

**Fig. 3.**
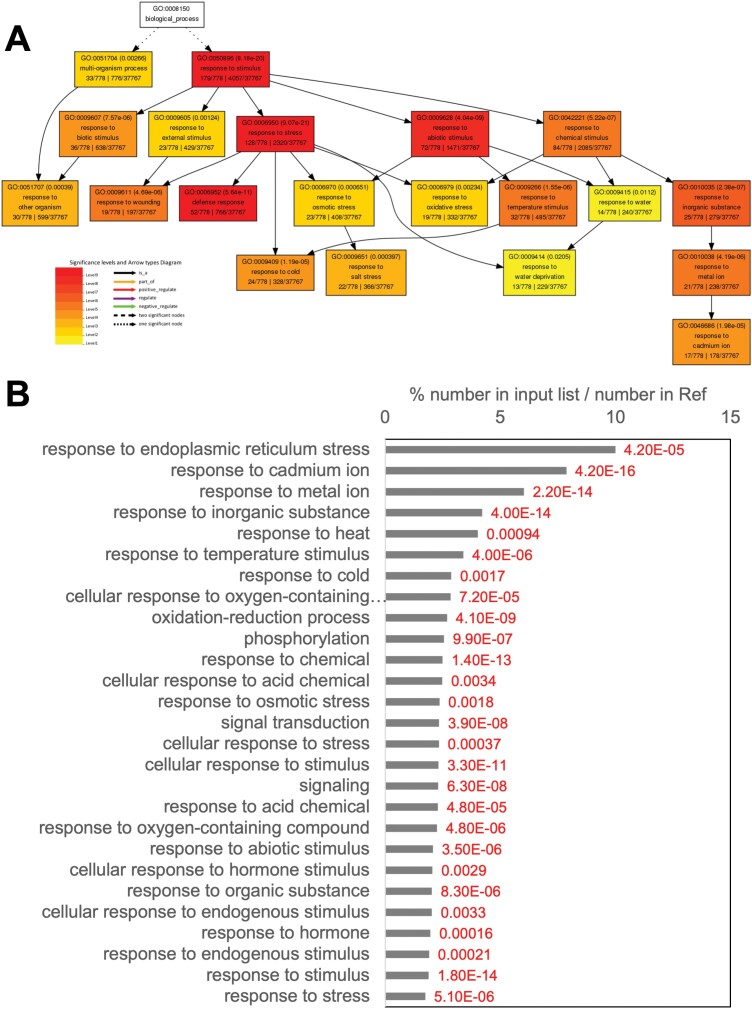
Gene Ontology (GO) enrichment. (A) GO enrichment of ABA-induced persulfidated proteins involved in response to stimulus. (B) GO enrichment of persulfidated targets in response to ABA susceptible to sulfenylation. The *P*-value for each GO term is annotated in red numbers.

Recently, a publication about ABA-triggered persulfidation of proteins (Laureano-Marín *et al*., 2020) revealed nearly 800 proteins that undergo persulfidation in response to ABA treatment in comparison with an untreated control (see Table S2 at Zenodo). Data can be obtained from ProteomeXchange Consortium via the PRIDE (Vizcaíno *et al.*, 2016) partner repository with the identifier PXD019802. The GO enrichment data of the persulfidated proteins induced by ABA treatment were processed using AgriGO (Table S3 at Zenodo). The GO term associated with response to stimulus, which contained 778 proteins, was analyzed to identify the most enriched GO terms (Fig. 3A), and it included 23 proteins in response to osmotic stress, 32 in response to temperature stimulus, 19 in response to oxidative stress, 24 in response to cold, 22 in response to salt stress, and 13 in response to water deprivation. In addition, another 52, 19, and 36 proteins were involved in defense response, wounding, and biotic stimulus, respectively. All the ABA-induced persulfidated proteins involved in abiotic stress are listed in Table S4 at Zenodo.

Overall, these results show that ABA treatment triggers persulfidation of a high number of proteins, and some of them aim to activate a cellular response to combat abiotic and biotic stresses. In addition, a total of 276 of these ABA-induced persulfidated proteins have been described as being sulfenylated. Further analysis of these targets shows that there are proteins involved in the response to abiotic stresses that are also susceptible to persulfidation and sulfenylation The GO enrichment data of the persulfidated proteins induced by ABA treatment, which can also be targets for sulfenylation, were processed using AgriGo tool (see Table S5 at Zenodo), and a selection of the GO-enriched terms associated with the stress response was constructed to identify the most enriched GO terms (Fig. 3B). Those GO terms most represented were response to endoplasmic reticulum stress (GO: 0034976) with a *P*-value of 0.000042 and a false discovery rate (FDR) of 0.00076, including six proteins in this GO term; response to cadmium (GO: 0046686) with scores of 4.2×10^–16^ and 8.8×10^–14^ for the *P*-value and FDR, respectively; and response to heat (GO: 0009408) and cold (GO: 0009409) with *P*-values of 0.00094 and 0.0017, and FDRs of 0.012 and 0.02, respectively. Nevertheless, as shown in Fig. 3B, other important GO terms, such as response to osmotic stress, signal transduction, and response to hormone are over-represented. These results highlight the existence of crosstalk between sulfenylation and persulfidation in response to certain abiotic stresses and that protein post-translational modifications play an important role in regulating these responses.

## Role of hydrogen sulfide in guard cell ABA signaling

As pointed out previously, the activation of ABA signaling pathways induces downstream targets that, in conjunction with ROS, Ca^2+^, and Ca^2+^-dependent protein kinases (CDPKs), activate ion channels to mediate stomatal closure and reduce water loss from transpiration (Mustilli *et al.*, 2002; Papanatsiou *et al.*, 2015). The participation of H_2_S in stomatal closure has also been described previously (García-Mata and Lamattina, 2010; Jin *et al.*, 2013). An initial study showed that ABA cannot induce the stomatal closure of *des* knockout mutants deficient in cytosolic DES1, which produces H_2_S in the cytosol (Alvarez *et al.*, 2010), while the addition of an exogenous H_2_S donor restored the closure. Moreover, ABA-dependent stomatal closure was partially blocked by an inhibitor of l-cysteine desulfhydrase and a scavenger of H_2_S, dl-propargylglycine (PAG) and hypotaurine (HT), respectively, suggesting that H_2_S participates in ABA-triggered stomatal movement (Scuffi *et al.*, 2014). Although DES1 is expressed at all growth stages, at the tissue level, green fluorescent protein (GFP) expression driven by the *DES1* promoter is very high in guard cells (Laureano-Marín *et al.*, 2014). It is also noteworthy that the *DES1* gene expression level in the RNA extracts of epidermal cells was several-fold higher than that in the mesophyll cell-enriched samples upon ABA treatment (Scuffi *et al.*, 2014), which provides a clue that the high expression level of *DES1* in epidermal cells may largely be due to the proportion of guard cells. Recently, genetic evidence also indicated that guard cell-specific expressed DES1 is required for *in situ* H_2_S production and is sufficient for regulating ABA-induced stomatal closure (Zhang *et al.*, 2020).

The synthesis of ABA is a central response to stress. Interestingly, guard cells contain the complete suite of ABA biosynthesis pathway components. The molybdenum cofactor sulfurase ABA3 that mediated ABA synthesis in guard cells is sufficient to induce stomatal closure and relieve leaf wilting (Bauer *et al.*, 2013). Several studies have revealed that H_2_S is involved in ABA synthesis. Exogenous application of NaHS was shown to increase the transcript levels of ABA biosynthesis-related genes during polyethylene glycol (PEG) treatment in both wheat leaves and wheat roots (Ma *et al.*, 2016). Consistently, it was found that the transcription of genes related to ABA biosynthesis, such as the 9-*cis*-epoxycarotenoid dioxygenases *NCED2*, *NCED3*, and *NCED5*, sharply increased in rice seedlings under drought stress conditions, and pre-treatment with NaHS further strengthened this inductive effect (Zhou *et al.*, 2020). Recently, Zhang *et al.* (2021a) demonstrated that the accumulation of all of the transcripts involved in ABA synthesis in leaves increased in the wild type but not in *des1* mutants, indicating that DES1 is essential for dehydration-induced ABA synthesis. Moreover, the H_2_S content was lower in the *aba3* mutant than in the wild type, suggesting that DES1-produced H_2_S is regulated by ABA synthesis. In addition, H_2_S participates in NO- and ethylene-induced stomatal closure (Hou *et al.*, 2013; Scuffi *et al.*, 2014).

Proteomic analyses have revealed that various proteins involved in ABA signaling are susceptible to persulfidation and that ABA treatments trigger the persulfidation of a considerable number of protein targets (Aroca *et al.*, 2017*a*; Laureano-Marín *et al*., 2020), thus suggesting that H_2_S regulates ABA signaling pathways through persulfidation of specific targets, including those within guard cells. In this way, the DES1-mediated guard cell ABA cascade is attributable to H_2_S signaling through persulfidation of open stomata 1 (OST1)/SNF1-RELATED PROTEIN KINASE 2.6 (SnRK2.6) at Cys131 and Cys137, which enhances ABA signaling (Chen *et al.*, 2020). Remarkably, SnRK2.6/OST1 is also nitrosylated by NO at Cys137, leading to the inhibition of its activity and further negatively regulating guard cell ABA signaling (Wang *et al.*, 2015). Crosstalk between H_2_S and NO has been previously described in the ABA signaling network in guard cells (Lisjak *et al.*, 2010; Scuffi *et al.*, 2014), and SnRK2.6/OST1 could be one of the targets driving this interplay.

DES1 itself is also activated by H_2_S through autopersulfidation at Cys44 and Cys205, which leads to transient H_2_S overproduction and the amplification of H_2_S signals in guard cells (Shen *et al.*, 2020). Activated DES1 persulfidates the NADPH oxidase RBOHD at Cys825 and Cys890 to rapidly induce a ROS burst that results in stomatal closure. Interestingly, persulfidation of both DES1 and RBOHD is redox dependent, and ROS accumulation at high levels induces persulfide oxidation, which inhibits the activity of these proteins, leading to ABA desensitization. The oxidized persulfides can be reduced back to thiol groups by thioredoxin and prevent the continuous activation of ABA signaling. Thus, these processes form a negative feedback loop through H_2_S- and ROS-mediated modification that finely tunes guard cell redox homeostasis and ABA signaling. In addition, the accumulation of ROS induced by H_2_S was also found to stimulate Ca^2+^ influx in guard cells (Wang *et al.*, 2016). Another element involved in guard cell DES-mediated ABA signaling has been recently defined: the transcription factor ABA insensitive 4 (ABI4). The DES1-dependent H_2_S accumulation induced by ABA generates the persulfidation of ABI4 at Cys250, promoting the MAPKKK18 transactivation, and thus propagating the mitogen-activated protein kinase (MAPK) signaling cascade in response to ABA (Zhou *et al.*, 2021a). Together, these findings hint at the complexity of the H_2_S signaling in stomatal movement.

The control of stomatal closure or opening relies on the activity of ion channels and ion transport proteins in the plasma and vacuolar membranes. The regulation of inward-rectifying K^+^ channels by H_2_S was shown in the sense that inactivation of the current associated with these channels induces stomatal closure by submicromolar concentrations of H_2_S (Papanatsiou *et al.*, 2015). Proof was also provided of activation by a low concentration of H_2_S of S-type anion currents in guard cells, the process of which requires elevated free cytosolic Ca^2+^ levels and OST1 function (Wang *et al.*, 2016). All these data highlight the complexity of the relationship between H_2_S and ion channels in the regulation of guard cell movement. Other secondary messengers that interact with H_2_S in the guard cell signaling network have been elucidated. In addition to the above-described ROS burst produced by NADPH oxidases, phospholipase D-derived phosphatidic acid is needed (Scuffi *et al.*, 2018; Liu *et al.*, 2021).

**Fig. 4.**
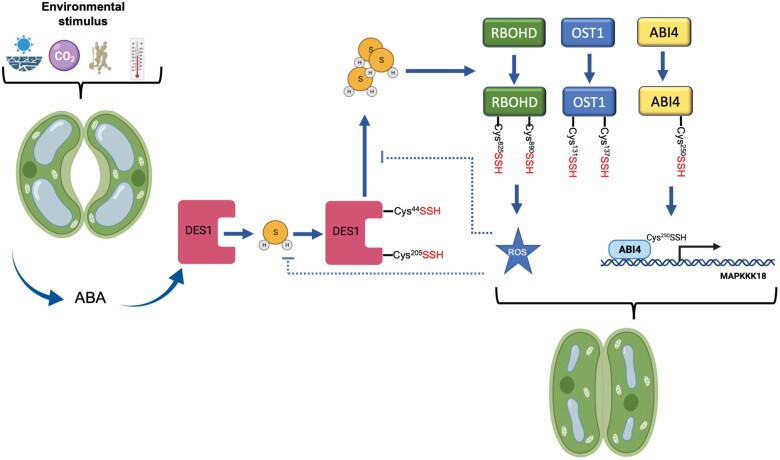
Graphical model of interconnections between H_2_S and ABA signaling networks in guard cells through the persulfidation of specific proteins. Under various environmental stress conditions, in guard cells, ABA concentrations increase and trigger the DES1 activity to induce the production of H_2_S to persulfidate specific protein targets. DES1 itself is persulfidated at Cys44 and Cys205, and causes the persulfidation of open stomata 1 (OST1) at Cys131 and Cys137, the NADPH oxidase RBOHD at Cys825 and Cys890, and ABI4 at Cys250. Persulfidated RBOHD produces a ROS burst that results in stomatal closure. Overaccumulation of ROS induces persulfide oxidation leading to ABA desensitivity. Persulfidated ABI4 promotes MAPKKK18 transactivation and MAPK signaling.

In summary, the H_2_S signaling network of stomatal movement is highly complex, and interactions among many different components of ABA-dependent signaling have been demonstrated (Pantaleno *et al.*, 2021). Moreover, accumulative evidence indicates that the H_2_S molecular mechanism involves persulfidation (Fig. 4).

## Conclusions and future perspectives

During past years, an immense number of plant studies describing the role of H_2_S in the regulation of essential processes have enabled H_2_S to be considered a signaling molecule of the same significance as NO and H_2_O_2_. Moreover, recent reports have permitted considerable insight into the molecular mechanism involved in H_2_S action in some specific processes such as ABA-dependent stomatal closure, and, importantly, to know the specific protein targets of persulfidation. Nevertheless, there is no doubt that the current challenges in the H_2_S field are, on one hand, to deepen knowledge of the molecular mechanism involved in H_2_S action and, on the other, to ascertain what is the bona fide sulfurating molecule that modifies the thiol group on proteins. Regarding the action of H_2_S, while an important effort has been made to establish chemical methods to label and enrich persulfidated proteins, and technical improvement of mass spectrometers have allowed the identification of an increasing number of plant proteins, mainly in Arabidopsis, knowledge of the H_2_S mechanism of action in a particular process is still scarce. As described above, insight into this mechanism has only been revealed to a certain extent in the ABA-dependent stomatal closure process, and in the regulation of autophagy by H_2_S, although, in the case of autophagy, the information is still limited.

With respect to the nature of the sulfurating species, this aspect is the subject of a great debate, even in animal systems. Due to the chemical nature of H_2_S, it cannot react directly with the thiols in proteins, and several scenarios leading to the formation of persulfidated proteins have been proposed. Thus, H_2_S can react with oxidized cysteine residues such as sulfenylated or nitrosylated cysteines or disulfides, and, therefore, under specific oxidative conditions, the sulfurating species can be H_2_S, or its ionic forms HS^−^ and S^2−^. Other sulfurating molecules proposed are polysulfides, which contain the form of sulfur named sulfane with the oxidation state of 0 and which have the ability to attach reversibly to other sulfur atoms (Ida *et al.*, 2014). We can hypothesize that depending on the specific condition/microenvironment of the target protein or the biological process in which the protein is involved, a particular sulfurating species or a mixture of them can be responsible for the protein persulfidation and it would be very difficult to differentiate between them. In addition, a prokaryotic and mammalian cysteinyl-tRNA synthetase has also been described that synthesizes persulfidated cysteine for direct incorporation to proteins (Akaike *et al.*, 2017). Another interesting point is to correlate the protein persulfidation pattern/level in one specific tissue/condition with the level of the sulfurating molecules. Perhaps in the context of a high level of persulfidation, it would be possible to discriminate which sulfurating species is responsible for performing persulfidation.

## Data Availability

A list of common proteins susceptible to persulfidation and sulfenylation, ABA-induced persulfidated proteins involved in abiotic stress, persulfidated proteins in response to ABA susceptible to *S*-sulfenylation, persulfidated proteins identified in response to ABA treatments, and GO enrichment of ABA-induced persulfidated proteins are available at Zenodo https://zenodo.org/record/4727058; Aroca *et al.* (2021*b*).

## Abbreviations

ABA: abscisic acid
CO: carbon monoxide
H_2_O_2_: hydrogen peroxide
NO: nitric oxide
ROS: reactive oxygen species
